# Hypothyroïdie congénitale à Dakar: à propos de 28 cas

**DOI:** 10.11604/pamj.2016.25.46.10321

**Published:** 2016-09-29

**Authors:** Babacar Niang, Amadou Lamine Fall, Idrissa Demba Ba, Younoussa Keita, Indou Dème Ly, Abou Ba, Aliou Thiongane, Aliou Abdoulaye Ndongo, Djibril Boiro, Lamine Thiam, Aissatou Ba, Morgiane Houngbadji, Mouhamed Fattah, Yaye Joor Djeng, Dieynaba Fafa Cissé, Idrissa Basse, Assane Sylla, Papa Moctar Faye, Saliou Diouf, Ousmane Ndiaye, Mamadou Sarr

**Affiliations:** 1 Département de Pédiatrie, Université Cheikh Anta Diop de Dakar, Sénégal; 2 Hôpital Aristide Le Dantec, CHU Dakar, Sénégal; 3 Hôpital d’Enfants de Diamniadio, Dakar, Sénégal; 4 Hôpital Abass Ndao, Dakar, Sénégal

**Keywords:** Hypothyroïdie congénitale, retard mental, retard de croissance, Congenital hypothyroidism, mental retardation, growth retardation

## Abstract

L’hypothyroïdie de l’enfant é été peu étudiée au Sénégal. Le but de cette étude était d’évaluer les aspects épidémiologiques, diagnostiques et évolutifs de l’hypothyroïdie congénitale. Il s’agissait d’une étude rétrospective descriptive et analytique portant sur tous les enfants suivis pour hypothyroïdie congénitale au Centre Hospitalier National d’Enfants Albert Royer sur la période de 2001 à 2014 (14 ans). A partir des dossiers des malades, nous avons recueilli et analysé les données sociodémographiques, cliniques et évolutives. Au total, 28 patients ont été inclus, soit une moyenne de 2 cas par an. L’âge moyen de découverte de l’hypothyroïdie était de 54,25 ± 43 mois avec une prédominance féminine (Sex- ratio 0,47). Seuls 2 cas d’hypothyroïdie ont été diagnostiqués dans la période néonatale. La consanguinité était présente chez 68% des patients. Les signes cliniques étaient dominés par le retard des acquisitions psychomotrices (96%), l’hypothermie (46%), la dysmorphie cranio-faciale (43%) et le goitre (39%). Le retard statural était constant au-delà de 6 mois. Les étiologies étaient dominées par les troubles de l’hormonosynthèse (84,21%). Dans l’évolution, la taille moyenne des patients était passée de -3,5 DS à -2,25 DS pour une durée de traitement moyenne de 28 mois. La débilité mentale était présente dans 73% des cas. Le retard de croissance et la débilité mentale étaient d’autant plus sévères que le diagnostic était tardif. Nos résultats confirment l’insuffisance d’une prise en charge précoce des patients. Il urge de mettre en place un système de dépistage néonatale systématique, afin d’améliorer le pronostic mental de cette affection.

## Abstract (en)

Child hypothyroidism has been little studied in Senegal. The aim of this study was to evaluate the epidemiological, diagnostic and evolutionary aspects of congenital hypothyroidism. We conducted a descriptive-analytical retrospective study of all children treated for congenital hypothyroidism at the Albert-Royer National Children’s Hospital Center over the period from 2001 to 2014 (14 years). We collected and analyzed socio-demographic, clinical and evolutionary data from patient medical records. A total of 28 patients were included in the study, an average of 2 cases per year. The average age of discovery of hypothyroidism was 54.25 ± 43 months with a female predominance (Sex-ratio 0.47). Only 2 cases of hypothyroidism were diagnosed in the neonatal period. Consanguinity was present in 68% of patients. Clinical signs were dominated by the delay in psychomotor acquisitions (96%), hypothermia (46%), cranio-facial dysmorphia (43%) and goiter (39%). Growth retardation was constant beyond 6 months. The etiologies were dominated by hormonosynthesis disorders (84.21%). During the study period, mean SD of patients had decreased from -3.5 SD to -2.25 SD for a median treatment duration of 28 months. Mental retardation was present in 73% of cases. Growth retardation and mental retardation were more severe as the diagnosis was late. Our results confirm the inadequacy of early management of patients. It is urgent to implement a routine neonatal screening system in order to improve the mental prognosis of this condition.

## Introduction

L’hypothyroïdie est l’expression clinique et biologique de la carence en hormones thyroïdiennes responsable d’un hypométabolisme, quelle que soit l’étiologie. Elle peut être congénitale ou acquise, permanente ou transitoire. L’hypothyroïdie congénitale (HC) représente la plus fréquente des endocrinopathies de l’enfant, concernant 1 naissance sur 3500 [1]. Il s’agit de la principale cause de retard mental évitable chez l’enfant [2]. Cependant dans notre contexte, elle n’est que très rarement diagnostiquée et il existe une sous-estimation de sa fréquence. En effet, en période néonatale, les manifestations cliniques sont discrètes et très peu caractéristiques. Le retard diagnostique qui en résulte, aboutit alors à un tableau de débilité mentale grave. La maladie n’est alors évoquée qu’à un stade tardif où le tableau clinique est caricatural. Dans les pays industrialisés, le recours depuis quelques décennies au dépistage systématique par le test sur papier buvard, a permet de reconnaître et de traiter précocement la grande majorité des HC. Ce dépistage ne se fait pas au Sénégal et dans la grande majorité des pays Africains. Le but de cette présente étude était d’évaluer les aspects épidémiologiques, diagnostiques et évolutifs de l’HC au Centre Hospitalier National d’Enfants Albert Royer (CHNEAR) de Dakar.

## Méthodes

Il s’agissait d’une étude rétrospective, descriptive et analytique, portant sur tous les dossiers d’enfants suivis pour hypothyroïdie congénitale biologiquement confirmée au CNHEAR sur une période de 14 ans (de 2001 à 2014). En l’absence de dépistage néonatale, le caractère congénital était évoqué sur la base de l’interrogatoire, si les symptômes débutaient avant l’âge de 6 mois. Pour chaque cas d’HC, nous avons recueilli, à partir des dossiers cliniques les paramètres suivants: les données socio-démographiques: âge de découverte, sexe, origine géographique, notion de consanguinité; Les données Cliniques: manifestations cliniques, développement psychomoteur, dysmorphie; La croissance: la taille prise en position couchée pour les enfants de moins de deux ans ou incapables de se tenir debout et en position debout pour les autres cas. Elle était exprimée en déviations standards (DS) selon les normes de croissance de l’OMS, permettant de définir: la croissance normale si la taille rapporté à l’âge était supérieure à -2 DS (entre -2 et +2DS); le retard de croissance modérée si la taille était inférieure à -2 DS mais supérieure à -3 DS pour l’âge; le retard de croissance sévère ou nanisme si la taille était inférieure à -3 DS pour l’âge; la taille cible pour les enfants sous traitement était inférieure ou égale à -1,5 DS.

Les paramètres radiologiques: âge osseux selon l’atlas de Greulich et Pyle sur des radiographies de la main et du poignet gauches ou du genou et du pied chez les nouveau-nés, morphologie taille et siège de la glande thyroïdienne à l’échographie thyroïdienne. Les paramètres biologiques (valeurs seuils de définition de l’hypothyroidie): T3 libre < 4 pmol/, T4 libre < 9 pmol/l; et TSH ultra-sensible > 4, 7 µIU/ml; Les données thérapeutiques: dose de L-thyroxine, délai d’euthyroïdie, croissance, devenir psychomoteur. Tous les paramètres ont été saisis à l’aide du logiciel Excel. Pour l’analyse descriptive les données étaient présentées en pourcentage pour les variables qualitatives et en moyennes avec Ecart-type pour les variables quantitatives. Les tests statistiques usuels utilisés étaient le Test de chi-2 pour les variables qualitatives et celui de Student pour les variables quantitatives. Un p < 0,05 était considéré comme statistiquement significatif avec un intervalle de confiance (IC) à 95%.

## Résultats

**Figure 1:**
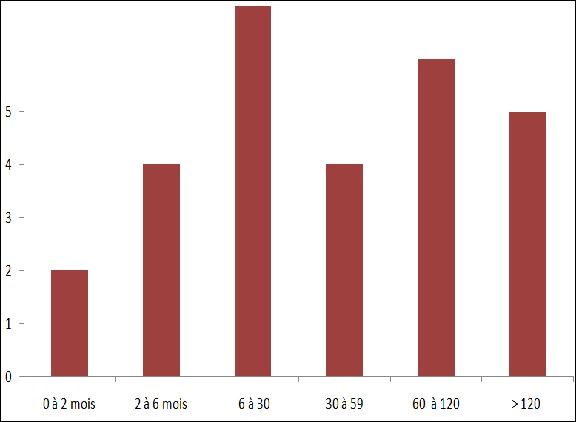
Répartition des patients en fonction de la tranche d’âge

**Sur le plan épidémiologique**: durant la période d’étude, 28 enfants ont été diagnostiqués et suivis pour une HC, soit en moyenne de deux cas par an. Ces cas d’HC représentaient 25,2% de l’ensemble des endocrinopathies (28/111). La **Figure 1** représente la répartition des cas d’HC en fonction de l’âge au diagnostic. L’âge moyen de découverte était de 54,25 ± 45 mois. La majorité (22 enfants soit 78,5%) avaient été diagnostiqués après l’âge de 6 mois. Deux cas seulement ont été diagnostiqués en période néonatale. Il y’avait une prédominance féminine (19 filles pour 9 garçons), soit un sex-ratio à 0,47. Une notion de consanguinité était retrouvée dans 68% des cas.

**Figure 2:**
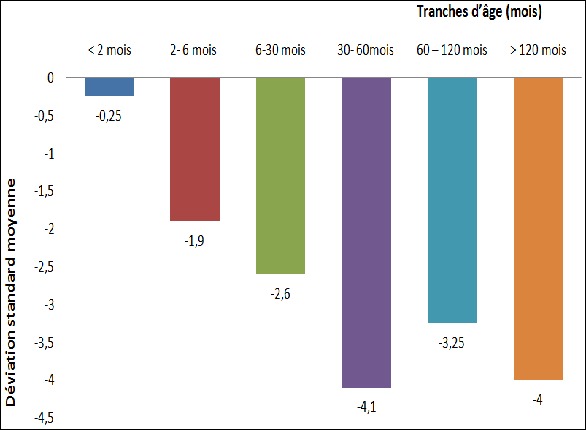
Sévérité du retard de croissance en fonction de l’âge de découverte

**Données cliniques:** Les signes cliniques au moment du diagnostic étaient dominés par le retard psychomoteur (26 cas soit 92,85%), la constipation (19 cas, soit 67,85%), l’hypothermie (13 cas, soit 46,42%), la dysmorphie cranio-faciale et la macroglossie (12 cas chacune, soit 42,85%). Les autres signes sont représentés dans le **Tableau 1**. L’évaluation de la croissance staturale avait permis d’objectiver 6 cas de croissance normale, tous âgés de moins de 6 mois, (soit 21,43%), 7 cas de retard de croissance modérée (25%) et 15 cas de nanisme (53,57%). La déviation standard moyenne (DS) était de -3,5. Le degré du retard statural était variable en fonction de l´âge au diagnostic: il était plus sévère pour les enfants âgés de plus de 30 mois au moment du diagnostic (p=0,002). Plus l’âge de découverte était tardif, plus le retard de croissance était sévère (**Figure 2**).

**Tableau 1:** Signes cliniques retrouvés au moment du diagnostic

Signes cliniques retrouves	Nombre	Pourcentage
Retard des acquisitions psychomotrices	26	92,85%
Constipation	19	67,85%
Hypothermie	13	46,42%
Macroglossie	12	42,85%
Dysmorphie cranio-faciale	12	42,85 %
Hernie ombilicale	11	39,28%
Goitre	11	39,28%
Hypotonie généralisée	8	28,57%
Troubles respiratoires	7	25%
Peau sèche infiltrée	5	17,85%

**Les signes radiologiques:** La maturation osseuse n’avait pu être évaluée que chez 15 enfants: un retard de l’âge osseux était noté dans 12 cas/15 (80%). L’âge osseux moyen (45 ± 41,84 mois) était inférieur à l’âge statural moyen (59,40 ± 43,62 mois) qui était inférieur à l’âge chronologique moyen (80,86 ±48,11 mois)

**Les signes échographiques:**Dix-huit échographies thyroïdiennes et une scintigraphie à l’iode^132^ ont été réalisées. L’orientation étiologique fondée sur ces résultats nous a permis de noter 16 cas de troubles de l’hormonogenèse (soit 84,21%) dont 11 cas sans goitre et 5 cas avec goitre et 3cas de troubles de la morphogenèse thyroïdienne (soit 15,79%) dont 1 cas d’ectopie et 2 cas d’hypoplasie thyroïdienne.

**Évolution:** Au cours du suivi, 23 enfants ont été régulièrement suivis, 4 ont été perdus de vue et 1 cas de décès à domicile de cause inconnue. Sous traitement, la déviation moyenne de la taille est passée de -3,5 à -2,25 pour une durée moyenne de traitement de 28 mois. Parmi les 17 patients régulièrement suivis et ayant un retard de croissance au moment du diagnostic, 4 (soit 23,53%) avaient atteint leur taille cible et 10 étaient passés d’un retard sévère à un retard modéré (58,82%). Seuls 3 cas de nanisme persistaient (17,65%). Le retard pubertaire a été noté chez 3 cas parmi les 6 ayant atteint l'âge de la puberté (50%) Le retard de développement psychomoteur était présent chez tous les 26 enfants âgés de plus de 2 mois au moment du diagnostic. Parmi eux 19 (73%) ont eu des séquelles définitives à type de débilité mentale de degré variable et seuls sept (7) ont été scolarisés avec retard. Parmi eux, les 6 ont été diagnostiqués avant 30 mois. Les deux enfants diagnostiqués à la période néonatale ont eu une croissance et un développement psychomoteur normaux. En moyenne, l’euthyroïdie biologique était obtenue en 5 mois avec une dose moyenne de 4,46 µg/kg/j de L thyroxine. Pour les cas d’hypoplasie thyroïdienne, l’hypothyroïdie persistait à des doses atteignant 10 µg/kg/j. Pour les troubles de l’hormonogenèse sans goitre, l’euthyroïdie était obtenue en 7 mois à la dose moyenne de 6 µg/kg/j alors que dans les troubles de l’hormonogenèse avec goitre, elle était obtenue en 2 mois à la dose moyenne de 3,8 µg/kg/j. La présence de goitre dans les troubles de l’hormonogenèse raccourcit le délai d’obtention de l’euthyroïdie (p=0,002) et réduit les besoins en L-thyroxine (p=0,005). Le **Tableau 2** résume les différentes caractéristiques des HC en fonction de l’étiologie.

**Tableau 2:** Caractéristiques des HC en fonction de l’étiologie

	Total (n= 28)	Absence d’échographie (n=9)	Dysmorphogenèse (n=3)	Dyshormonogenèse sans goitre (n=11)	Dyshormonogenèse avec goitre (n=5)
Sexe (F/M)	19/9	5/4	3/0	7/4	2/3
Age moyen	54,25 mois	-	16 mois	66 mois	78 mois
T4L initiale	4,17pmol/l	-	Indétectable	2,87 pmol/l	5,4 pmol/l
TSHus initiale	68,80mIU/l	-	>100mIU/l	76,4 mIU/l	54 mIU/l
Délai euthyroïdie	5 mois	-	Pas d’euthyroïdie stable	7 mois	2 mois
Dose L-thyroxine	4,46 µg/kg/j	-	10µg/kg/j	6µg/kg/j	3,8 µg/kg/j

TSH us: thyréostimuline hypophysaire ultra-sensible, T4: tétraiodothyronine

## Discussion

La faible fréquence de l’hypothyroïdie retrouvée dans notre étude est probablement liée à une absence de dépistage systématique qui, seule, permet d’avoir une estimation réelle de la prévalence de cette affection. Dans les pays développés, l’incidence est passée de 1 sur 10000 à 1 cas sur 3500 à 4000 naissances avec l’avènement du dépistage systématique dans les années 1970 [1, 3]. L’âge de découverte est tardif. Le même constat a été fait au Sénégal par Fall en 1986 [4] et en Tunisie par Hachicha en 2002 [5]. Ce qui met en jeu le pronostic mental. La prédominance féminine corrobore les résultats précédemment décrits au Sénégal par Fall, en 1986. Aussi en Afrique, Hachicha retrouvait une prédominance féminine. De même, en Europe, la prédominance féminine a été largement démontrée [3, 6]. Cependant, en 2015, Barry Y montrait que cette prédominance féminine concernait plutôt les hypothyroïdies liées à une dysgénésie alors que dans les hypothyroïdies avec glande en place, le sex-ratio était équilibré [7]. Sur le plan étiologique, les troubles de l’hormonosynthèse prédominaient avec 84,21% des causes. Dans la littérature occidentale, les dysgénésies thyroïdiennes sont responsables de 70 à 80% des hypothyroïdies congénitales et les troubles congénitaux d’hormonogenèse ne sont responsables que de 10 à 20% des hypothyroïdies congénitales [1, 8, 9]. Par contre en Afrique, les troubles de l’hormonosynthèse semblent dominer les causes. Ainsi, elles étaient responsables de 66,7% des causes au Sénégal [4], de 48% des causes en Tunisie [5] et de 7cas sur 8 au Cameroun [10]. Ceci est probablement lié à la fréquence des unions consanguines, les troubles de l’hormonosynthèse étant liés à des maladies génétiques à transmission autosomique récessive. Dans notre série, nous avons retrouvé 68% de cas de consanguinité. L’évolution est marquée par des séquelles à type de débilité mentale et de retard de croissance. Le mauvais pronostic était lié au retard diagnostique et probablement aux doses de L-thyroxine utilisées. En effet, la dose moyenne utilisée chez nos patients était de 4,46 µg/kg/j et selon les données récentes, des doses de 10- 15µg/kg/j sont nécessaires pour le traitement de l’hypothyroïdie congénitale [11]. Des études ont montré qu’une dose initiale de L-thyroxine insuffisante est associée au mauvais pronostic [11, 12]. Les dysgénésies étaient plus sévères que les troubles de l’hormonosynthèse. En effet selon les auteurs, les dysgénésies étaient plus sévères et requièrent de plus forte dose de L-thyroxine [11–13]. Près des 3/4 de nos patients présentaient des séquelles neurologiques incompatibles avec une scolarisation, du fait du retard diagnostique. D’où l’importance de mettre en place un système de dépistage systématique. Dans les pays développés où ce système de dépistage est mis place, le diagnostic précoce et le traitement approprié a transformé le pronostic notamment neurologique de cette affection [12–14].

## Conclusion

Les hypothyroïdies dans nos contextes sont caractérisées par un retard diagnostique. Ce retard est associé à un mauvais pronostic mental et statural. D’où l’intérêt d’un diagnostic précoce et notamment le dépistage systématique qui, seul permet des données fiables sur l’épidémiologie et d’améliorer le pronostic de cette affection. Les étiologies sont dominées par les troubles de l’hormonosynthèse, ceci constitue une piste de recherche quant à la prédominance ou non de cette dernière dans les causes d’hypothyroïdie congénitale en Afrique.

### Etat des connaissances actuelles sur le sujet

- L’hypothyroïdie congénitale est l’endocrinopathie congénitale la plus fréquente avec une incidence d’environ 1:3000 naissances en Occident;
- Il s’agit de la cause de retard mental évitable grâce au dépistage néonatal systématique.

### Contribution de notre étude à la connaissance

- Très peu de données africaines sont disponibles dans la littérature cette étude reflète les difficultés de prise en charge l’hypothyroïdie congénitale dans un pays sous développé;
- L’âge de découverte est tardif par rapport à celui dans les pays développés;
- Les troubles de l’hormonosynthèse dominent les causes, et semblent moins sévères comparés aux troubles de la morphogenèse qui constituent la principale cause en occident.
